# Feeding cats with chronic kidney disease food supplemented with betaine and prebiotics increases total body mass and reduces uremic toxins

**DOI:** 10.1371/journal.pone.0268624

**Published:** 2022-05-24

**Authors:** Jean A. Hall, Dennis E. Jewell, Eden Ephraim

**Affiliations:** 1 Department of Biomedical Sciences, College of Veterinary Medicine, Oregon State University, Corvallis, Oregon, United States of America; 2 Department of Grain Science and Industry, Kansas State University, Manhattan, Kansas, United States of America; 3 Pet Nutrition Center, Hill’s Pet Nutrition, Topeka, Kansas, United States of America; Universidade de Trás-os-Montes e Alto Douro: Universidade de Tras-os-Montes e Alto Douro, PORTUGAL

## Abstract

Cats with chronic kidney disease (CKD) have a decreased ability to maintain body weight. As CKD advances, loss of body weight contributes to morbidity and mortality. The goal of this study was to evaluate the combined effects of feeding betaine and prebiotics on body weight of both CKD and healthy cats. The pre-trial food (control food) was a complete and balanced dry food designed to aid in the management of CKD. Test food was the control food supplemented with betaine (0.500%) and prebiotics: long-chain oat beta-glucan (0.586%) and 0.407% short chain fructooligosaccharides (scFOS). The CKD cats (n = 7) were fed pre-trial food for 28 days and then randomly assigned to control food or test food. Each food was fed for 8 weeks in a cross-over study design. In a second study, healthy cats received control food or test food for 8 weeks (n = 8 each group). Blood, urine, and fecal samples were collected to evaluate concentrations of relevant kidney function biomarkers and metabolites at the end of each feeding period for CKD cats, and blood samples were collected monthly to evaluate concentrations of plasma metabolites for healthy cats. Body weight and composition were measured using dual-energy X-ray absorptiometry (DEXA) scan at baseline and after each feeding period. Total body mass was significantly higher in CKD cats after consuming test food compared with control food (*P* = 0.004), with no significant difference in food intake while consuming test or control food (*P* = 0.34). Test food did not affect total body mass or composition of healthy cats. Indole compounds produced by bacterial metabolism were decreased in urine and increased in feces of CKD cats fed test food, and plasma concentrations were negatively correlated with the level of kidney function, indicating a potential benefit of consuming test food. In healthy cats, consuming test food resulted in significantly decreased concentrations of plasma P-cresol sulfate (*P* = 0.004) and increased concentrations of docosahexaenoic acid (DHA) and eicosapentaenoic acid (EPA; both *P* < 0.05), despite the fact that both control and test foods had similar concentrations of these long-chain fatty acids, 0.03% and 0.02%, respectively. These results suggest that the addition of betaine and prebiotics to the control food formula may have increased total body mass in CKD cats by enhancing one-carbon metabolism and by modulating the gut microbiome.

## Introduction

Aging in cats is associated with sarcopenia [1]. We have previously shown that total lean mass decreases with increasing age in otherwise healthy cats [1]. Cats with chronic kidney disease (CKD) also have a decreased ability to maintain body weight. As CKD advances, loss of body weight contributes to morbidity and mortality [2].

In humans, no reliable interventions currently exist to prevent CKD induced muscle wasting, although mechanisms that increase loss of cellular protein have been identified [3]. For example, the decline in kidney function noted in aging human populations may be associated with increased oxidative stress and inflammation [4]. Food is a major source of oxidants, and foods can be modified to effect changes in oxidant burden [4]. Thus, innovative strategies can be developed to suppress protein loss based on nutrition.

The goal of this study was to evaluate the combined effects of feeding betaine and prebiotics on body weight of both CKD and healthy cats. The ability of prebiotics to modulate the gut microbiome has been well documented [5, 6]. In human CKD patients, prebiotics favor the growth of saccharolytic bacteria [7] that produce short chain fatty acids beneficial to the host. Simultaneously, they decrease the proportion of proteolytic bacteria that produce toxic nitrogenous wastes [8]and decrease kidney function (reviewed in [9]). Prebiotics also have been reported to increase nutrient absorption [10].

The prebiotics used in these studies were short-chain fructo-oligosaccharides (scFOS) and long-chain oat beta-glucan. The scFOS prebiotic is a well-defined fiber that is quickly fermented. Oat beta-glucan is a natural soluble fiber. It is a viscous polysaccharide made up of repeating glucose subunits with bonds that are beta 1–3 and beta 1–4 linkages. The beta 1–3 linkages make it soluble. In comparison, the indigestible fiber cellulose is also a beta glucan, but it is insoluble because it has only beta 1–4 linkages.

Betaine is known to have osmoprotective effects on the kidney [11]. It also decreases concentrations of homocysteine by serving as a methyl donor [12]. In addition, betaine acts as a chaperone to stabilize protein structure under denaturing conditions [13].

Neither betaine nor prebiotics are known to increase body weight in humans [14, 15]. We conducted two studies to evaluate the combined effects of feeding betaine and prebiotics on body weight of cats. In the first study, our goal was to investigate the effects of feeding renal-protective foods, supplemented with functional food bioactives, to cats with International Renal Interest Society (IRIS) stage 1 and 2 CKD, to determine if such foods increase or maintain lean body mass and improve negatively altered biomarkers and metabolites associated with aging. In the second study, healthy cats were fed the same food to determine if the effects noted in CKD cats were similar for healthy cats. We assessed plasma, urine, and fecal metabolite concentrations, and in particular, markers of inflammation (e.g., sphingolipid metabolites [16, 17]) and oxidative stress (e.g., those involved in tocopherol and glutathione metabolism). The main finding of this study was that feeding a combination of betaine and prebiotics increased body mass in CKD cats compared with feeding control food.

## Methods

All study protocols were reviewed and approved by the Institutional Animal Care and Use Committee, Hill’s Pet Nutrition, Inc., Topeka, KS, USA (Permit Numbers: CP609.0.0.0-A-F-D-ADH-MULTI-162-KID and P711.0.0.0-A-F-D-ADH-MULTI-67-WTM), and complied with the National Institutes of Health Guide for the Care and Use of Laboratory Animals [18]. Cats were housed in groups and allowed access to indoor runs. Cats also had exposure to natural light that varied with seasonal changes. All cats were provided with regular opportunities to exercise, with access to toys. Cats were owned by the commercial funders of this research or their affiliates, who gave permission for them to be included in this study. At the conclusion of the study, all cats were returned to the Hill’s Pet Nutrition, Inc. colony.

### Participants and study design

**Fig 1:**
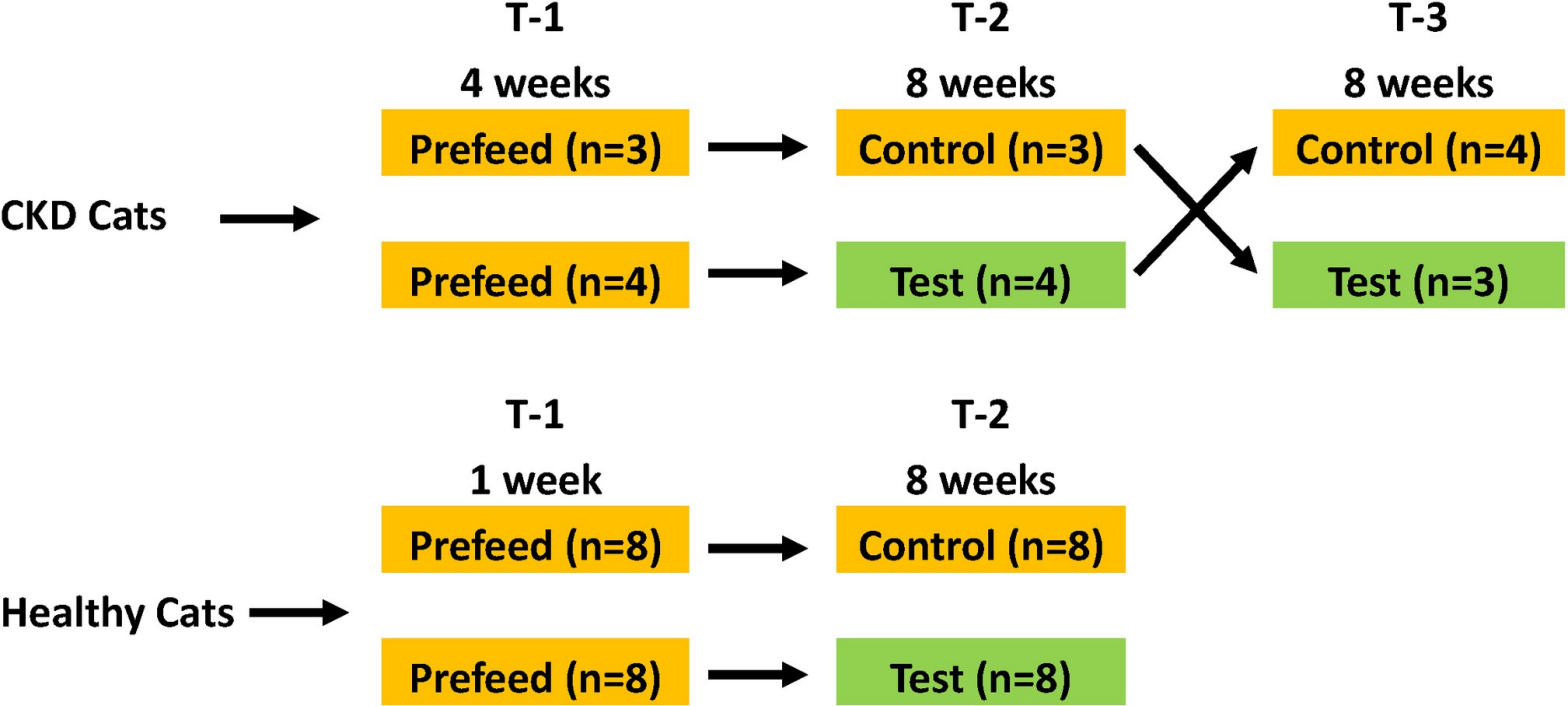
Study design. Pre-trial food and control food were the same. Test food was the pre-trial food supplemented with betaine (0.500%) and prebiotics: oat beta-glucan (0.586%) and scFOS (0.407%).

Two studies were conducted to evaluate the effect of naturally occurring dietary ingredients on kidney health (**Fig 1**). The first study was a crossover trial with seven CKD cats. All cats were fed either test food or control food for 8 weeks and then were crossed over to the other food for 8 weeks. The study used a pre-trial food (control food) and a test food that contained betaine and prebiotics: long-chain oat beta-glucan and short-chain fructo-oligosaccharides (scFOSs). The second study was a randomized study with 16 healthy cats assigned to control or test food. Each group of eight cats received control or test food for 8 weeks. Control and test food were the same in both studies.

The pre-trial food (control food) was a complete and balanced dry food designed to aid in the management of CKD. Test food was the pre-trial food supplemented with betaine (0.500%) and prebiotics: oat beta-glucan (0.586%) and scFOS (0.407%). The CKD cats were fed pre-trial food for 4 weeks in study one and then were randomly assigned to control food or test food in a cross-over study design. In the second study, all 16 healthy cats were fed the pre-trial food for one week, as an independent study showed that one week was an adequate wash out period in healthy cats fed test food similar to their off-test food [19]. The cats were then randomly assigned to the control food (eight cats) or the test food (eight cats). Healthy cats received control food or test food for 8 weeks. Because the total number of cats in the second study was greater, a cross-over study was not needed. All cats had access to electronic feeders whereby fresh food was offered daily with amounts available for consumption calculated to maintain body weight; water was available *ad libitum*. Actual daily food intake (g/day) was recorded for each cat.

All cats were of domestic shorthair breed. At baseline, cats in the CKD study ranged in age from 6.0 to 16.2 years, and body weights ranged from 3.8 to 7.1 kg. There were four spayed females and three neutered males. Inclusion criteria were cats with CKD or kidney stones. Cats were considered reasonable to have CKD if one or more of these diagnostic findings were observed: 1) persistently increased serum creatinine (Cr ≥1.6 mg/dL), 2) persistently increased serum symmetric dimethylarginine (SDMA > 14 μg/dL), or 3) persistently dilute urine specific gravity (USG ≤ 1.030) without identifiable non-renal cause. This included cats with CKD (IRIS stages 1 and 2; n = 3; one had kidney stones), cats with kidney stones (n = 3), and one cat with a missing right kidney. Cats with CKD were assigned IRIS stage at baseline based on the results of an annual physical examination, complete blood count (CBC), serum biochemistries, and urinalysis [20]. Cats were excluded from the group if they were known to have problems eating new foods or problems with repeat blood sampling, and/or had any other diagnosed disease condition such as diabetes, cancer, inflammatory bowel disease, dermatitis, or food allergy. Cats in the second study were healthy cats of ideal body weight and with no evidence of CKD or other diseases. Cats were determined to be healthy based on the results of an annual physical examination, CBC, serum biochemistries, and urinalysis. At baseline, healthy cats ranged in age from 2.6 to 9.3 years, and body weights ranged from 3.2 to 5.8 kg. There were 11 spayed females and five neutered males. The criterion for removal from either study was development of any condition whereby removal would benefit the animal, including any cat refusing to eat, or inadequate food intake resulting in weight loss greater than 15% of body weight. No cats were removed from either study.

Blood, urine, and fecal samples were collected at baseline (end of pre-trial period) and at the end of each 8-week feeding period to evaluate changes in plasma, urine, and fecal metabolites in CKD cats. Assays also included CBC, serum biochemical analysis, urinalysis, and urine protein/creatinine (UPC) ratio. Body weight and composition were measured using dual-energy X-ray absorptiometry (DEXA) scan at baseline and after each feeding period. For healthy cats, blood was collected at baseline, at one month into the feeding trial, and at the end of the eight-week feeding period.

### Foods

A complete and balanced dry food designed to aid in the management of CKD, was fed during the pre-trial period and was the control food. Test food was the pre-trial food supplemented with betaine (0.500%) and prebiotics: oat beta-glucan (0.586%) and scFOS (0.407%). Oat beta-glucan was 22% beta glucan (0.129%); scFOS was 95% scFOS (0.387%) and thus, 3× higher in concentration than oat beta-glucan. Both foods (**Table 1**) were prepared by Hill’s Pet Nutrition, Inc., and met the nutritional requirements for adult cats (≥ 1 year) as established by the Association of American Feed Control Officials (AAFCO). Food was available in dry form only. Macronutrient composition and fiber concentrations of foods were determined by a commercial laboratory (Eurofins Scientific, Inc., Des Moines, IA). Proximate analyses were completed using the following techniques: moisture—AOAC 930.15; protein—AOAC 2001.11; fat—AOAC 954.02; fiber—AOAC 962.09; and ash—AOAC 942.0. Mineral and fatty acid analyses were performed by the same commercial laboratory. Fatty acid (FA) concentrations were determined by gas chromatography of FA methyl esters, and were expressed as g/100 g of FAs as fed. The sum of dietary saturated FA (SFA) was determined as follows: 8:0+10:0+11:0+12:0+14:0+15:0+16:0+17:0+18:0+20:0+22:0+24:0. The sum of dietary monounsaturated FA (MUFA) was determined as follows: 14:1+15:1+16:1+17:1+18:1+20:1+22:1+24:1. The sum of dietary polyunsaturated FA (PUFA) was determined as follows: 18:2(n-6)+18:3(n-6)+18:3(n-3)+18:4(n-3)+20:2(n-6)+20:3(n-6)+20:3(n-3)+20:4(n-6)+20:4(n-3)+20:5(n-3)+21:5(n-3)+22:2(n-6)+22:4(n-6)+22:5(n-6)+22:5(n-3)+22:6(n-3).

**Table 1:** Composition of pre-trial (Control food^1^) and test food^2^.

Nutrient	Pre-trial & Control Food	Test Food
Moisture	4.98	5.34
Protein	28.0	28.0
Fat	19.4	19.5
Atwater Energy,^3^ kcal/kg	4083	4077
Ash	4.27	4.42
Crude Fiber	1.8	1.6
Calcium	0.73	0.72
Phosphorus	0.52	0.51
Sodium	0.22	0.22
ARA [20:4 (n-6)]	0.09	0.09
EPA [20:5 (n-3)]	0.02	0.02
DHA [22:6 (n-3)]	0.03	0.03
SFA^4^	6.35	6.51
MUFA^5^	7.51	7.80
PUFA^6^	3.69	3.81
Total FA	17.64	18.31
(n-6) FA^7^	3.50	3.60
(n-3) FA^8^	0.21	0.23
(n-6):(n-3) ratio	16.7	15.7

^1^Pre-trial (control) food was a complete and balanced dry food designed to aid in the management of CKD. All analytical values are expressed as percentage of food, as fed, unless otherwise indicated.

^2^ Test food was prepared by Hill’s Pet Nutrition, Inc. and was similar to the pre-trial food, with the exception that it was supplemented with betaine (0.500%) and prebiotics: oat beta-glucan (0.586%) and scFOS (0.407%).

^3^ Energy was calculated using the modified Atwater factors as described [21].

^4^ Sum of the SFA: 8:0+10:0+11:0+12:0+14:0+15:0+16:0+17:0+18:0+20:0+22:0+24:0.

^5^ Sum of the MUFA: 14:1+15:1+16:1+17:1+18:1+20:1+22:1+24:1.

^6^ Sum of the PUFA: 18:2(n-6)+18:3(n-6)+18:3(n-3)+18:4(n-3)+20:2(n-6)+20:3(n-6)+20:3(n-3)+20:4(n-6)+20:4(n-3)+20:5(n-3)+21:5(n-3)+22:2(n-6)+22:4(n-6)+22:5(n-6)+22:5(n-3)+22:6(n-3).

^7^ Sum of the (n-6) fatty acids.

^8^ Sum of the (n-3) fatty acids.

Both foods contained similar concentrations (within analytical variance of targets) of protein, and had similar predicted caloric content. Likewise, crude fiber and fatty acid composition were similar for control and test foods. Test food had added betaine at 0.500% and prebiotics: oat beta-glucan (0.586%) and short-chain fructooligosaccharides (scFOS) (0.407%). Rice was used as the carbohydrate source and reduced from the control food (42.3 to 40.7%) to allow for these additions. Foods protein sources were corn gluten meal (19.4% in control, 19.5% in test food to balance the protein contained in the rice that was replaced), poultry by-product meal (9%), egg (3%), spray dried chicken (2%) and fish meal (1.4%). Fat was added as pork fat (14.4%) and fiber was added to both foods through cellulose (1.8%) and beet pulp (0.6%). Both foods received similar amounts of vitamins, amino acids and minerals.

### Chemical analyses for biomarkers and metabolites: Plasma, urine, and fecal metabolomics

Analysis of plasma (CKD and healthy cats) and urine (CKD cats only) metabolomic profiles was performed by a commercial laboratory (Metabolon, Morrisville, NC) as previously described [22]. Briefly, extracted supernatant was split and run on gas chromatography and liquid chromatography mass spectrometer platforms in randomized order. Gas chromatography (for hydrophobic molecules) and liquid chromatography (for hydrophilic molecules) were used to identify and provide relative quantification of small metabolites present in plasma and urine samples. Endogenous biochemical included amino acids, peptides, carbohydrates, lipids, nucleotides, cofactors and vitamins. The complete datasets are shown as a heat map of statistically significant biochemicals profiled in this study (**S1–S3 Tables**).

Fecal samples (CKD cats only) were collected, homogenized, and frozen as aliquots within 1 hour of defecation. Whole feces were collected after defecation and homogenized thoroughly using Thinky Mixer model ARM-310 (THINKY USA, Inc). Homogenous samples were aliquoted into labeled cryovials. The tubes were snap-frozen immediately in liquid nitrogen followed by storing at −80°C until further processing. Extracted supernatants were prepared for fecal metabolomics as described for plasma and urine, and the dataset is reported (**S4 Table**).

### Statistical methods

Statistical analyses were performed in JMP, version 15 (SAS Institute, Cary, NC) for food intake and body weights. These data were normally distributed. The CBC, serum chemistries, urinalyses and metabolomics data were natural log transformed before analyses. Metabolomics data were analyzed using Array Studio (OmicSoft Corporation, Cary NC). Matched-paired *t* tests were used for the CKD cats to compare means taken on each cat after the consumption of test and control foods. An ANOVA mixed model was used to analyze the data for healthy cats. Significance was established when *P* ≤ 0.05 (for type 1 error) and *q* ≤ 0.1 (*q*-values were used to estimate false discovery rate in multiple comparisons).

## Results

### CKD cats

Total body mass was significantly higher in CKD cats after consuming test food compared with control food (*P* = 0.004), as all 7 CKD cats had higher total body mass after consuming test food (**Table 2**). All CKD cats had higher total fat mass (*P* = 0.17) and 6 of 7 cats had higher lean body mass (*P* = 0.14) after consuming test food, however these were not significantly different compared with after consuming control food. Total bone mass was not different between CKD cats after consuming test and control food (*P* = 0.34). There was no significant difference in food intake while consuming test or control food in CKD cats (*P* = 0.34), although both test (*P* <0.0001) and control (*P* = 0.0002) food intakes were higher compared with baseline food intake.

**Table 2:** Total body mass and food intake before (Baseline) and after feeding test and control foods for eight weeks in Chronic Kidney Disease (CKD) cats and healthy cats^1^.

Cat group	Total body Mass (g)	Total fat Mass (g)	Total Lean Mass (g)	Total Bone Mass (g)	Food Intake (g/day)	Food Intake (kcal/day)
**CKD cats**						
Baseline	5399.5 ± 410.4	1699.9 ± 234.8	3554.3 ± 298.4	145.3 ± 9.3	41.8 ± 0.9^b^	169.1 ± 3.6^b^
Control food	5175.8 ± 410.3^b^	1516.6 ± 211	3538.8 ± 314	145.2 ± 9.9	45.7 ± 0.6^a^	184.9 ± 2.4^a^
Test food	5346.9 ± 412.2^a^	1584.4 ± 214.3	3590.9 ± 300	146.8 ± 10.4	46.6 ± 0.7^a^	188.1 ± 2.7^a^
**Healthy cats**						
Baseline	5142.4 ± 267.2	1177.8 ± 101.9	3831.4 ± 183.1	147.3 ± 7	45.1 ± 2.2^b^	182.3 ± 9.1^b^
Control food	4999.2 ± 345.5	1123.9 ± 191.8	3775 ± 195.6	142.3 ± 9.8	56.7 ± 0.95^a^	229.1 ± 3.9^a^
Test food	5377.9 ± 415.1	1527.6 ± 160.6	3786.4 ± 295.1	156.3 ± 11.1	59.6 ± 0.95^a^	240.4 ± 3.8^a^

^1^Results are shown as mean ± SEM.

^a,b^Means with different superscripts within a column indicate significant differences between cats consuming test and control food, in either the CKD or healthy cat study, or significant differences between baseline and test or control food consumption.

Cats were considered reasonable to have CKD if one or more of these diagnostic findings were observed: 1) persistently increased creatinine (Cr ≥1.6 mg/dL), 2) persistently increased symmetric dimethylarginine (SDMA > 14 μg/dL), or 3) persistently dilute urine specific gravity (USG ≤ 1.030) without identifiable non-renal cause. Cats with CKD included cats that were persistently azotemic with Cr >1.6 mg/dL over an extended period, typically for ≥ 3 months (n = 3); one of these cats had nephrolithiasis), nonazotemic cats with kidney stones (n = 3), as well as a nonazotemic cat with a missing kidney based on physical examination and ultrasonographic imaging (n = 1; right kidney missing). Proteinuria was classified as borderline proteinuric if urine protein/creatinine (UPC) ratio was 0.2 to 0.4, and proteinuric if UPC ratio was > 0.4. The three azotemic cats were borderline proteinuric at baseline. One nonazotemic cat with kidney stones became azotemic and proteinuric after consuming control and test foods. No CKD cats were removed from the study. A matched-paired t test using natural log transformed values was performed to determine differences between cats in serum and urine kidney function biomarkers after consuming test vs control food, and differences within CKD cats from baseline vs after consuming control food, and from baseline vs after consuming test food (**Table 3**). Only USG for baseline vs after consuming test food was significantly different (decreased *P* = 0.046) for CKD cats.

**Table 3:** Serum and urine kidney function biomarkers before (Baseline) and after feeding control and test food for eight weeks in Chronic Kidney Disease (CKD) cats.

Cat group	BUN (mg/dL)	Cr (mg/dL)	SDMA (ug/dL)	UPC	USG
**CKD cats**					
Baseline^1^	27.3 ± 2.6	1.6 ± 0.1	14.9 ± 1.4	0.2 ± 0.05	1.032 ± 0.006^a^
Control food^1^	51.0 ± 25.2	3.9 ± 2.3	35.4 ± 20.2	0.3 ± 0.12	1.032 ± 0.006
Test food^1^	26.0 ± 1.8	1.6 ± 0.1	16.1 ± 1.0	0.2 ± 0.06	1.029 ± 0.005^b^
Baseline vs Control^2^	0.24 ± 0.27	0.3 ± 0.32	0.38 ± 0.27	0.16 ± 0.4	0.0005 ± 0.002
Baseline vs Test^2^	-0.04 ± 0.04	-0.007 ± 0.03	0.09 ± 0.04	-0.026 ± 0.27	-0.004 ± 0.003
Test vs Control^2^	-0.28 ± 0.29	-0.3 ± 0.33	-0.3 ± 0.3	-0.18 ± 0.15	-0.004 ± 0.002

^1^Results are shown as mean ± SEM.

^2^Results are *mean difference* ± SEM of the natural logs

^a,b^Means with different superscripts within a column indicate significant differences between baseline and after consuming test or control food.

BUN = blood urea nitrogen; Cr = creatinine; SDMA = symmetric dimethyl arginine; UPC = urine protein/creatinine ratio; USG = urine specific gravity

Plasma metabolites in CKD cats that were significantly different after consuming control food vs test food are shown in **Table 4**. Betaine, dimethylglycine, sarcosine, and methionine were increased with test food consumption. Multiple metabolites associated with phospholipid metabolism (and a lysolipid) were decreased after consuming test food compared with control food, whereas fatty acid metabolism (palmitoylcholine) was increased. The anti-oxidative product of vitamin E metabolism, gamma-tocopherol/beta-tocopherol, also was increased after cats consumed test food compared with control food.

**Table 4:** Relative concentrations of plasma metabolites that were significantly different (*P* ≤ 0.05) in cats (N = 7) with Chronic Kidney Disease (CKD) after feeding control food or test food for 8 weeks in a crossover feeding trial.

Plasma Metabolites	Mean Values^1^		*P*-value
	Control Food	Test Food	
**Glycine, Serine and Threonine Metabolism**			
Sarcosine	0.80	1.04	0.027
Dimethylglycine	0.86	1.45	0.001
Betaine	0.79	1.72	0.000
**Alanine and Aspartate Metabolism**			
N-methylalanine	1.09	1.73	0.024
**Tryptophan Metabolism**			
Indoleacetylglutamine	0.59	0.95	0.019
**Methionine, Cysteine, SAM and Taurine Metabolism**			
Methionine	0.91	1.14	0.026
**Phospholipid Metabolism**			
1-palmitoyl-2-oleoyl-GPC (16:0/18:1)	1.02	0.95	0.037
1-stearoyl-2-arachidonoyl-GPE (18:0/20:4)	1.02	0.82	0.009
1-palmitoyl-2-arachidonoyl-GPE (16:0/20:4)	1.13	0.80	0.000
1-palmitoyl-2-linoleoyl-GPE (16:0/18:2)	1.05	0.75	0.004
1-stearoyl-2-linoleoyl-GPE (18:0/18:2)	1.08	0.89	0.043
1-linoleoyl-2-arachidonoyl-GPC (18:2/20:4n6)	0.94	1.08	0.047
1-linoleoyl-2-arachidonoyl-GPE (18:2/20:4)	1.13	0.80	0.048
**Lysolipid**			
1-palmitoyl-GPE (16:0)	1.03	0.85	0.031
**Fatty Acid Metabolism (Acyl Choline)**			
Palmitoylcholine	0.74	0.96	0.027
**Tocopherol Metabolism**			
Gamma-tocopherol/beta-tocopherol	0.87	1.07	0.013

^1^For each metabolite, mean value is the group mean of re-scaled data to have medial equal to 1.

Control food was a complete and balanced dry food formulated for cats to aid in the management of CKD. Test food was similar to control food, with the exception that it was supplemented with betaine (0.500%) and prebiotics: oat beta-glucan (0.586%) and scFOS (0.407%).

Urine metabolites in CKD cats that were significantly different after consuming control food vs test food are shown in **Table 5**. Concentrations of ketose and associated metabolites were increased after consuming test food, whereas indole compounds were decreased including 3-indoxyl sulfate, 5-hydroxyindole sulfate, 6-hydroxyindole sulfate, and 7-hydroxyindole sulfate. Betaine, dimethylglycine, and sarcosine were increased with test diet. The concentrations of many other amino acid catabolites also were altered in the urine. Mevalonate excretion was decreased in urine after consuming test food.

**Table 5:** Relative concentrations of urine metabolites that were significantly different (*P* ≤ 0.05) in cats with Chronic Kidney Disease (CKD) after feeding control food or test food for 8 weeks in a crossover feeding trial (N = 7).

Urine Metabolites	Mean Values^1^		*P*-value
	Control Food	Test Food	
**Glycine, Serine and Threonine Metabolism**			
Sarcosine	0.53	1.24	0.020
Dimethylglycine	0.77	2.13	0.021
Betaine	1.03	1.41	0.013
**Glutamate Metabolism**			
Glutamine	1.23	0.97	0.031
N-acetylglutamine	1.27	0.84	0.003
**Tryptophan Metabolism**			
3-indoxyl sulfate	1.25	0.90	0.063
5-hydroxyindole sulfate	0.99	0.62	0.027
6-hydroxyindole sulfate	1.06	0.64	0.037
7-hydroxyindole sulfate	1.14	0.73	0.023
**Leucine, Isoleucine and Valine Metabolism**			
3-methylcrotonylglycine	1.01	0.80	0.048
Valine	1.00	0.83	0.024
**Methionine, Cysteine, SAM and Taurine Metabolism**			
N-methyltaurine	0.98	2.24	0.001
**Acetylated Peptides**			
Phenylacetylserine	0.76	1.09	0.006
**Fructose, Mannose and Galactose Metabolism**			
Mannitol/sorbitol	1.03	1.20	0.042
Galactonate	1.20	0.75	0.004
**Fatty Acid, Monohydroxy**			
3-hydroxysebacate	1.14	1.39	0.002
7-hydroxyoctanoate	1.05	0.60	0.027
**Phospholipid Metabolism**			
Glycerophosphoinositol	1.29	0.67	0.029
**Lysolipid**			
1-palmitoleoyl-GPC (16:1)	0.36	0.43	0.023
**Mevalonate Metabolism**			
Mevalonate	0.98	0.26	0.009
Mevalonolactone	1.11	0.74	0.017
**Primary Bile Acid Metabolism**			
Tauro-beta-muricholate	0.53	0.20	0.050
**Food Component/Plant**			
1-kestose	0.26	1.49	0.001
1,1-kestotetraose	0.47	0.90	0.028
3-hydroxyindolin-2-one	0.80	0.59	0.016
Betonicine	1.22	0.78	0.008
Glucoheptose	1.23	0.96	0.031
Homostachydrine	1.22	0.74	0.010
Stachydrine	1.03	0.71	0.028

^**1**^For each metabolite, mean value is the group mean of re-scaled data to have medial equal to 1.

Control food was a complete and balanced dry food formulated for cats to aid in the management of CKD. Test food was similar to control food, with the exception that it was supplemented with betaine (0.500%) and prebiotics: oat beta-glucan (0.586%) and scFOS (0.407%).

Fecal metabolites in CKD cats that were significantly different after consuming control food vs test food are shown in **Table 6**. Many amino acid catabolites were altered in feces after consuming test food. For example, metabolites from aromatic amino acid catabolism were increased (4-hydroxyphenylacetate, indolelactate, 2,4,6-trihydroxybenzoate, and daidzein). Carnosine and anserine were increased in feces with consumption of test food. Syringic acid was also increased in the feces after consuming test food. Lipid associated changes included increased levels of fatty acids (dicarboxylate), lysolipids, glycerophosphoglycerol, diacylglycerols, and glycolipid metabolites, although some PUFAs, monoacylglycerol, and sphingolipid metabolites were decreased.

**Table 6:** Relative concentrations of fecal metabolites that were significantly different (*P* ≤ 0.05) in cats with Chronic Kidney Disease (CKD) after feeding control food or test food for 8 weeks in a crossover feeding trial (N = 7).

Fecal Metabolites	Mean Values^1^		*P*-value
	Control Food	Test Food	
**Histidine Metabolism**			
Histamine	0.15	0.21	0.038
N-acetylhistamine	0.31	0.42	0.009
**Phenylalanine and Tyrosine Metabolism**			
4-hydroxyphenylacetate	0.52	1.71	0.005
**Tryptophan Metabolism**			
Indolelactate	0.77	1.51	0.044
Thioproline	0.79	1.22	0.039
**Methionine, Cysteine, SAM and Taurine Metabolism**			
N-acetylcysteine	0.59	1.19	0.019
**Urea cycle; Arginine and Proline Metabolism**			
N-delta-acetylornithine	1.04	1.51	0.014
**Glutathione Metabolism**			
Cysteinylglycine	0.78	1.38	0.011
**Gamma-glutamyl Amino Acid**			
Gamma-glutamylalanine	1.39	0.83	0.016
Gamma-glutamylmethionine	1.24	0.89	0.048
**Dipeptide Derivative**			
Carnosine	0.90	1.21	0.000
Anserine	0.85	1.16	0.028
**Aminosugar Metabolism**			
N-acetylglucosamine 6-sulfate	0.55	1.89	0.023
**TCA Cycle**			
Fumarate	1.54	0.85	0.050
**Polyunsaturated Fatty Acid (n3 and n6)**			
Dihomo-linolenate (20:3n3 or n6)	1.33	0.75	0.008
**Fatty Acid, Dicarboxylate**			
2-hydroxyadipate	0.70	1.03	0.000
Sebacate (decanedioate)	0.99	1.24	0.040
**Lysolipid**			
1-stearoyl-GPC (18:0)	0.89	1.37	0.004
1-stearoyl-GPE (18:0)	0.83	1.32	0.023
**Glycerolipid Metabolism**			
Glycerophosphoglycerol	0.58	1.10	0.025
**Monoacylglycerol**			
2-palmitoylglycerol (16:0)	0.95	0.59	0.024
**Diacylglycerol**			
Oleoyl-arachidonoyl-glycerol (18:1/20:4) [2]	0.92	1.26	0.045
Stearoyl-linoleoyl-glycerol (18:0/18:2) [2]	0.70	1.11	0.018
**Sphingolipid Metabolism**			
N-butyroyl-sphingosine (d18:1/4:0)	1.00	0.51	0.022
**Glycolipid Metabolism**			
1,2-dilinoleoyl-galactosylglycerol (18:2/18:2)	0.92	1.00	0.029
**Pyrimidine Metabolism, Uracil containing**			
2’-deoxyuridine	0.88	1.57	0.019
**Pyrimidine Metabolism, Thymine containing**			
Thymidine	0.88	1.44	0.009
Thymine	1.05	1.56	0.045
**Benzoate Metabolism**			
2,4,6-trihydroxybenzoate	0.94	1.16	0.030
**Food Component/Plant**			
Daidzein	0.62	0.96	0.048
Syringic acid	0.66	1.14	0.018

^**1**^For each metabolite, mean value is the group mean of re-scaled data to have medial equal to 1.

Control food was a complete and balanced dry food formulated for cats to aid in the management of CKD. Test food was similar to control food, with the exception that it was supplemented with betaine (0.500%) and prebiotics: oat beta-glucan (0.586%) and scFOS (0.407%).

In the CKD cats, plasma uremic toxins (all indole sulfate metabolites) were positively correlated (*P* ≤ 0.05) to plasma kidney biomarkers (SDMA and creatinine) and urine uremic toxins (again all indole sulfate metabolites) as shown in **Table 7**.

**Table 7:** Significant (*P* ≤ 0.05) correlations for plasma uremic toxins and plasma kidney biomarkers and urine uremic toxins in cats (N = 7) with Chronic Kidney Disease (CKD) after feeding control food or test food for 8 weeks in a crossover feeding trial.

Plasma Uremic Toxins	Correlate	P-value	r
3-hydroxyindolin-2-one sulfate	Blood SDMA	0.001	0.78
3-hydroxyindolin-2-one sulfate	Blood Creatinine	0.001	0.79
3-hydroxyindolin-2-one sulfate	Urine 3-hydroxyindolin-2-one sulfate	0.034	0.57
3-hydroxyindolin-2-one sulfate	Urine 3-indoxyl sulfate	0.048	0.54
3-hydroxyindolin-2-one sulfate	Urine 6-hydroxyindole sulfate	0.049	0.53
3-indoxyl sulfate	Blood SDMA	0.006	0.69
3-indoxyl sulfate	Blood Creatinine	0.006	0.70
3-indoxyl sulfate	Urine 3-indoxyl sulfate	0.018	0.62
3-indoxyl sulfate	Urine 6-hydroxyindole sulfate	0.019	0.62
3-indoxyl sulfate	Urine 3-hydroxyindolin-2-one sulfate	0.022	0.61
3-indoxyl sulfate	Urine 5-hydroxyindole sulfate	0.048	0.54
5-hydroxyindole sulfate	Blood SDMA	0.001	0.78
5-hydroxyindole sulfate	Blood Creatinine	0.001	0.79
5-hydroxyindole sulfate	Urine 3-indoxyl sulfate	0.043	0.55
6-hydroxyindole sulfate	Blood SDMA	0.033	0.57
6-hydroxyindole sulfate	Blood Creatinine	0.030	0.58
6-hydroxyindole sulfate	Urine 6-hydroxyindole sulfate	0.005	0.70
6-hydroxyindole sulfate	Urine 3-indoxyl sulfate	0.006	0.69
6-hydroxyindole sulfate	Urine 3-hydroxyindolin-2-one sulfate	0.006	0.69
6-hydroxyindole sulfate	Urine 5-hydroxyindole sulfate	0.015	0.63
6-hydroxyindole sulfate	Urine 7-hydroxyindole sulfate	0.030	0.58
7-hydroxyindole sulfate	Blood SDMA	0.018	0.62
7-hydroxyindole sulfate	Blood Creatinine	0.018	0.62
7-hydroxyindole sulfate	Urine 3-hydroxyindolin-2-one sulfate	0.011	0.65
7-hydroxyindole sulfate	Urine 3-indoxyl sulfate	0.016	0.97
7-hydroxyindole sulfate	Urine 6-hydroxyindole sulfate	0.017	0.63
7-hydroxyindole sulfate	U_5-hydroxyindole sulfate	0.026	0.59

Control food was a complete and balanced dry food formulated for cats to aid in the management of CKD. Test food was similar to control food, with the exception that it was supplemented with betaine (0.500%) and prebiotics: oat beta-glucan (0.586%) and scFOS (0.407%).

### Healthy cats

In the healthy cats, none of the DEXA measurements for total body mass, total fat mass, total lean mass, or total bone mass were different between cats consuming test or control foods, nor different from baseline values (**Table 2**). Although numerically food intake in the healthy cats was greater while consuming test food compared with control food, the difference was not significant (*P* = 0.35). Food intakes of both test and control foods were greater compared with baseline food intake (*P* <0.0001) for healthy cats.

Plasma metabolites in healthy cats that were significantly different after consuming control food vs test food are shown in **Table 8**. Similar to CKD cats, increased levels of betaine, dimethylglycine, and cysteine were noted in cats consuming test food. The most pronounced signatures following the ingestion of the test food came from lipid metabolism. These included increased plasma PUFAs, phospholipids, phosphatidylcholine metabolites, sphingolipids, and ceramides. Consumption of test food resulted in a significant increase in circulating levels of the long-chain fatty acids: EPA, DPA, DHA, and AA, despite the fact that both control and test foods had similar levels of EPA, DHA, and AA.

**Table 8:** Relative concentrations of plasma metabolites that were significantly different (*P* ≤ 0.05) in healthy cats after feeding control food (N = 8) or test food (N = 8) for 8 weeks.

Plasma Metabolites	Mean Values^1^		*P*-value
	Control Food	Test Food	
**Glycine, Serine and Threonine Metabolism**			
Dimethylglycine	0.85	1.63	0.000
Betaine	1.01	2.60	0.000
**Histidine Metabolism**			
Imidazole propionate	1.01	1.62	0.020
Imidazole lactate	0.90	1.47	0.000
N-acetylhistamine	1.78	0.66	0.019
**Tyrosine Metabolism**			
Phenyllactate (PLA)	0.90	1.40	0.027
Phenol sulfate	1.56	0.78	0.037
**Methionine, Cysteine, SAM and Taurine Metabolism**			
Cysteine	0.88	1.05	0.024
**Urea cycle; Arginine and Proline Metabolism**			
Arginine	0.90	1.07	0.006
Ornithine	0.91	1.10	0.022
Trans-4-hydroxyproline	1.14	0.78	0.003
**Polyunsaturated Fatty Acid (n3 and n6)**			
Heneicosapentaenoate (21:5n3)	0.76	1.40	0.023
Eicosapentaenoate (EPA; 20:5n3)	0.96	1.83	0.049
Docosapentaenoate (n3 DPA; 22:5n3)	0.87	1.42	0.019
Docosahexaenoate (DHA; 22:6n3)	0.91	1.56	0.006
Arachidonate (20:4n6)	0.98	1.45	0.007
**Fatty Acid, Dicarboxylate**			
Octadecanedioate	1.03	1.36	0.028
Eicosanodioate	0.89	1.29	0.008
**Fatty Acid Metabolism (also BCAA Metabolism)**			
Propionylglycine	1.31	0.88	0.045
**Phospholipid Metabolism**			
Choline	1.06	1.19	0.030
Glycerophosphorylcholine (GPC)	1.02	1.34	0.008
Phosphoethanolamine	1.15	1.54	0.010
**Phosphatidylcholine (PC)**			
1,2-dipalmitoyl-GPC (16:0/16:0)	0.95	1.18	0.035
1-palmitoyl-2-oleoyl-GPC (16:0/18:1)	1.03	1.21	0.046
1-palmitoyl-2-arachidonoyl-GPC (16:0/20:4n6)	0.96	1.25	0.006
1-palmitoyl-2-docosahexaenoyl-GPC (16:0/22:6)	0.94	1.43	0.001
1-stearoyl-2-arachidonoyl-GPC (18:0/20:4)	0.94	1.18	0.003
1-linoleoyl-2-arachidonoyl-GPC (18:2/20:4n6)	0.93	1.37	0.014
1-stearoyl-2-docosahexaenoyl-GPC (18:0/22:6)	0.92	1.43	0.002
1-oleoyl-2-docosahexaenoyl-GPC (18:1/22:6)	0.97	1.56	0.003
**Phosphatidylinositol (PI)**			
1-stearoyl-2-linoleoyl-GPI (18:0/18:2)	1.16	0.76	0.002
**Lysophospholipid**			
1-lignoceroyl-GPC (24:0)	0.99	1.42	0.030
2-stearoyl-GPE (18:0)	1.31	0.89	0.049
**Sphingolipid Metabolism**			
N-palmitoyl-sphinganine (d18:0/16:0)	0.98	1.71	0.030
Myristoyl dihydrosphingomyelin (d18:0/14:0)	0.80	1.32	0.004
Behenoyl sphingomyelin (d18:1/22:0)	0.93	1.38	0.039
Sphingomyelin (d18:1/14:0, d16:1/16:0)	0.79	1.20	0.005
Sphingomyelin (d18:2/14:0, d18:1/14:1)	0.81	1.25	0.030
Sphingomyelin (d17:1/16:0, d18:1/15:0, d16:1/17:0)	0.93	1.37	0.016
Sphingomyelin (d18:2/16:0, d18:1/16:1)	0.97	1.28	0.028
Sphingomyelin (d18:1/21:0, d17:1/22:0, d16:1/23:0)	0.87	1.25	0.038
Sphingomyelin (d18:1/22:1, d18:2/22:0, d16:1/24:1)	0.88	1.13	0.034
Sphingomyelin (d18:2/23:0, d18:1/23:1, d17:1/24:1)	0.92	1.29	0.020
Sphingomyelin (d18:1/24:1, d18:2/24:0)	0.97	1.31	0.037
Sphingosine	1.06	1.44	0.019
Sphingosine 1-phosphate	1.05	1.25	0.030
Sphingomyelin (d18:2/23:1)	0.91	1.34	0.007
Sphingomyelin (d18:2/21:0, d16:2/23:0)	0.88	1.30	0.023
Sphingomyelin (d18:1/20:2, d18:2/20:1, d16:1/22:2)	0.93	1.24	0.022
Sphingomyelin (d17:2/16:0, d18:2/15:0)	0.87	1.46	0.005
Sphingomyelin (d18:2/18:1)	1.05	1.35	0.047
Sphingomyelin (d18:1/19:0, d19:1/18:0)	0.93	1.37	0.025
**Ceramides**			
N-palmitoyl-sphingosine (d18:1/16:0)	0.99	1.37	0.019
Ceramide (d18:1/14:0, d16:1/16:0)	0.80	1.39	0.033
Ceramide (d18:2/24:1, d18:1/24:2)	0.95	1.32	0.028
**Tocopherol Metabolism**			
alpha-tocopherol	0.97	1.14	0.075
**Benzoate metabolism**			
Catechol sulfate	1.39	0.80	0.054
4-methylcatechol sulfate	2.22	0.75	0.010
Methyl-4-hydroxybenzoate sulfate	0.18	2.41	0.000
p-cresol sulfate	1.67	0.74	0.004
**Food Component/Plant**			
Equol sulfate	1.44	0.51	0.003
Stachydrine	1.10	0.85	0.034
Pyrraline	1.20	0.67	0.021

^**1**^For each metabolite, mean value is the group mean of re-scaled data to have medial equal to 1.

Control food was a complete and balanced dry food formulated for cats to aid in the management of CKD. Test food was similar to control food, with the exception that it was supplemented with betaine (0.500%) and prebiotics: oat beta-glucan (0.586%) and scFOS (0.407%).

The healthy cats in the test group had significantly lower levels of plasma P-cresol sulfate, catechol sulfate, and 4-methylcatechol sulfate after consumption of test food containing the combination of betaine and prebiotics. Consuming test food also led to reduced levels of plasma trans-4-hydroxyproline, a marker of collagen degradation, and to reduced levels of the advanced glycation end product (AGE), pyrraline.

## Discussion

The significant finding of this study was that feeding CKD cats a combination of dietary betaine and prebiotics resulted in increased body mass compared with feeding control food. The addition of betaine and prebiotics (oat beta-glucan and scFOS) to the control food resulted in a higher body mass in CKD cats independent of food intake. In finishing mini-pigs, dietary betaine has been shown to increase average daily gain and final body weight, while reducing average backfat thickness and increasing lean percentage [23]. Betaine may have the most influence on growth under conditions of metabolic or nutritional stress [24]. For example, in men undergoing strength training, betaine supplementation for a 6-week period improved body composition (lean body mass and fat mass) and bench press work capacity [24]. In the latter study it was hypothesized that betaine supplementation enhances protein synthesis, and thus, improves body composition by reducing homocysteine and homocysteine thiolactone [24]. Because homocysteine impairs insulin signaling by reducing insulin receptor substrate-1 activation, it inhibits Akt-phosphorylation [25]. If CKD cats consumed excess methionine and inadequate folate and betaine, homocysteine transmethylation may be impaired resulting in excess homocysteine thiolactone. Although homocysteine thiolactone was not detected in urine in our study, this mechanism deserves further investigation in future studies. In our study, CKD cats did have higher plasma concentrations of betaine and methionine after consuming test food for 8 weeks.

Unlike its effect on CKD cats, test food did not significantly affect total body mass or body composition of healthy cats. Although food intake in healthy cats consuming both control food and test food were increased compared with baseline food intake, similar food intake was observed for both treatments, and there was no effect of test food on total body mass. Therefore, this study suggests that the unique effects of test food on body weight is specific to CKD cats. This suggests that betaine and prebiotics aid in the management of CKD by imparting an increased ability to utilize food.

The results of the metabolomics data for plasma, urine, and feces of CKD cats provided evidence that test food contained prebiotics. Prebiotic metabolites were increased in urine and other carbohydrates linked with microbial activity were increased in feces after consuming test food. Prebiotics are non-digestible food ingredients that selectively stimulate the growth and/or activity of specific bacteria in the colon with the intent to improve host health. The test diet contained prebiotics that influenced bacterial carbohydrate metabolism. The 1,1-kestotetraose and 1-kestose are from inulin-type fructans, a prebiotic that encourages the colonization of beneficial bacteria. Ketose and associated metabolites in the urine of cats consuming test food are produced by lactic acid producing bacteria such as bifidobacterial and lactobacilli.

Amino acid metabolites produced exclusively by microflora were altered in CKD cats after consuming test food, which also suggested changes in the microfloral activity. Intestinal bacteria contribute to the metabolism of aromatic amino acids, and related metabolites were increased in feces. Indole compounds produced by bacterial metabolism were decreased in urine of CKD cats consuming test food indicating a potential benefit of consuming test food. For example, indole is produced from tryptophan by gut microflora and subsequently converted to indoxyl sulfate in the liver. Indoxyl sulfate in the plasma was negatively correlated with the level of kidney function in CKD cats. Thus, these changes could reflect improved microflora activity, gut permeability, or kidney function.

Healthy cats consuming test food had reduced plasma levels of P-cresol sulfate. P-cresol is a by-product of microbial fermentation of aromatic amino acids such as tyrosine and phenylalanine by gut bacteria. In the liver, it is converted to P-cresol sulfate to be removed by the kidney. P-cresol sulfate is a uremic toxin known to deteriorate kidney function. Both indoxyl sulfate and P-cresol sulfate have been shown to induce inflammation and oxidative stress [26] suggesting a potential benefit of the test food for healthy aging cats as well.

Other changes that reflect microflora metabolism include mevalonate and carnosine/anserine excretion and one-carbon metabolism. Bacteria use the mevalonate pathway to synthesize ubiquinone for the electron transport chain and dolicol-P for cell wall peptidoglycan synthesis. Mevalonate excretion was decreased in urine of CKD cats consuming test food suggesting that the utilization of the mevalonate pathway by bacteria had been altered by the test food.

Betaine is an important nutrient that can support methyl donation reactions to provide protective biochemicals for stressful conditions. Because test food was supplemented with 0.500% betaine, it was not surprising that betaine was increased in plasma and urine with consumption of test food. This provided enhanced capacity for one-carbon metabolism, evidenced by increased dimethylglycine, sarcosine, and methionine (downstream metabolites of betaine). For example, a methyl donation from betaine to homocysteine would increase methionine, and theoretically S-adenosylmethionine (SAM) and S-adenosylhomocysteine (SAH).

Intact betaine is a low molecular weight compound that accumulates in the cell cytoplasm in order to protect the structure of proteins and enzymes under abiotic stress (serves as a bioprotectant) [27]. The primary role of betaine in the kidney is osmoprotection of cells of the medulla [28]. Betaine has a strong ability to bind a considerable amount of water and this hydration plays an important role in its stabilizing effect on proteins. Otherwise, cell dehydration would result in protein denaturation and loss of enzyme biological activity. Thus, higher plasma levels of betaine in CKD and healthy cats after consuming test food is advantageous as it would increase the tolerance of cells to unfavorable conditions.

Anserine and carnosine were increased in feces after consumption of test food further supporting that one-carbon metabolism was enhanced. Anserine and carnosine are histidine derived dipeptides with antioxidant capacity. Beta-alanine is combined with histidine to generate the dipeptide carnosine, which then can be converted to anserine by N-methylation at the imidazole ring with SAH acting as the methyl donor.

In healthy cats, higher levels of betaine and one-carbon metabolites in cats fed test food confirmed dietary efficacy. Increased plasma choline and phosphatidylcholine metabolites were also suggestive of increased circulating lipoproteins. Choline is required for a wide range of biological activities, including providing the methyl groups in a number of biosynthetic reactions in the form of betaine, maintaining the structural integrity of cell membranes in the form of phosphatidylcholine, and supporting cholinergic neurotransmission in the form of acetylcholine. The healthy cats consuming test food had higher levels of choline compared with cats consuming control food. It is possible that increased betaine availability in the test food freed up choline for phosphatidylcholine synthesis instead of its conversion to betaine. Phosphatidylcholine can also be synthesized from phosphatidylethanolamine through the action of phosphatidylethanolamine N-methyltransferase using SAM as the methyl donor, which comes from the downstream action of betaine. Collectively, the levels of phosphatidylcholine can be impacted by betaine supplementation via different routes.

The most pronounced signatures following ingestion of the test food in healthy cats involved lipid metabolism, including increased circulating phospholipids, sphingolipids, and free fatty acids. The synthesis of sphingomyelin from ceramide involves phosphatidylcholine, whereas the release of ceramide from sphingomyelin frees up a choline phosphate. The levels of various ceramides, sphingomyelins, and associated sphingolipids all were increased in cats consuming test food relative to control food. Notably, many of these metabolites such as sphingosine, sphingosine 1-phosphate, and ceramide are bioactive lipids that are involved in lipid signaling. Increased circulating PUFAs were also observed in the test food group and may be reflective of increased mobilization from triacylglycerol and/or decreased fatty acid beta-oxidation. Because markers of fatty acid beta-oxidation did not reveal any changes, the former is more plausible.

Consuming test food resulted in significant increases in plasma docosahexaenoic acid (DHA) and eicosapentaenoic acid (EPA) in healthy cats, two important n-3 FAs, despite the fact that both control and test foods had similar levels, 0.03% and 0.02%, respectively. Increased uptake or reduced degradation may explain these changes in plasma DHA and EPA. Higher levels of DHA and EPA would be beneficial (reviewed in [29, 30]) as increases are reflected in greater incorporation into blood lipid, cell and tissue pools. This in turn can modify the structure of cell membranes and also the function of membrane proteins involved as receptors, signaling proteins, transporters, and enzymes. These very long-chain n-3 fatty acids also modify the production of lipid mediators and through effects on cell signaling can alter patterns of gene expression.

Decreased markers of collagen degradation were also observed in cats consuming test food. Collagen is the most abundant protein in the extracellular matrix, and free trans-4-hydroxyproline is almost exclusively found in collagen. Degradation of collagen by the enzyme matrix metalloproteinases gives rise to this metabolite. Decreased levels of trans-4-hydroxyproline, thus, may reflected decreased extracellular matrix breakdown. Markers of collagen degradation have been linked to kidney fibrosis [31]. The reduction of plasma trans-4-hydroxyproline in cats implies improved collagen integrity and kidney health.

Pyrraline is an advance glycation end product (AGE) that was reduced in healthy cats after consuming test food. Because AGEs are associated with increased oxidative stress and are known to increase in cats with CKD [32] as well as with aging [33] this represents another positive benefit for cats with the addition of betaine and prebiotics (oat beta-glucan and scFOS) to the control food formula.

## Conclusions

These results show a benefit of feeding betaine and prebiotics to CKD cats to aid in the management of CKD by increasing body mass without increasing food consumption. An increase in plasma betaine metabolites supports enhanced capacity for one-carbon metabolism. Amino acid catabolites were beneficially altered in urine and feces of CKD cats after consuming test food suggesting altered microbiota activity. Although body mass was not increased after feeding betaine and prebiotics to healthy cats, test food was shown to minimize the accumulation of uremic toxins and to increase the concentrations of DHA and EPA, both of which support healthy aging and wellbeing in cats.

## Supporting information

S1 Table Heat map of statistically significant plasma biochemicals from CKD cats profiled in this study. Red and green shaded cells indicate p≤0.05 (red indicates that the mean values are significantly higher for that comparison; green values significantly lower). Light red and light green shaded cells indicate 0.05<p<0.10 (light red indicates that the mean values trend higher for that comparison; light green values trend lower). For the ANOVA, blue-shaded cells indicate p≤0.05; light blue-shaded cells indicate 0.05<p<0.10.
(XLSX)

S2 Table Heat map of statistically significant urine biochemicals from CKD cats profiled in this study. Red and green shaded cells indicate p≤0.05 (red indicates that the mean values are significantly higher for that comparison; green values significantly lower). Light red and light green shaded cells indicate 0.05<p<0.10 (light red indicates that the mean values trend higher for that comparison; light green values trend lower). For the ANOVA, blue-shaded cells indicate p≤0.05; light blue-shaded cells indicate 0.05<p<0.10.
(XLSX)

S3 Table Heat map of statistically significant plasma biochemicals from healthy cats profiled in this study. Red and green shaded cells indicate p≤0.05 (red indicates that the mean values are significantly higher for that comparison; green values significantly lower). Light red and light green shaded cells indicate 0.05<p<0.10 (light red indicates that the mean values trend higher for that comparison; light green values trend lower). For the ANOVA, blue-shaded cells indicate p≤0.05; light blue-shaded cells indicate 0.05<p<0.10.
(XLSX)

S4 Table Heat map of statistically significant fecal biochemicals from CKD cats profiled in this study. Red and green shaded cells indicate p≤0.05 (red indicates that the mean values are significantly higher for that comparison; green values significantly lower). Light red and light green shaded cells indicate 0.05<p<0.10 (light red indicates that the mean values trend higher for that comparison; light green values trend lower). For the ANOVA, blue-shaded cells indicate p≤0.05; light blue-shaded cells indicate 0.05<p<0.10.
(XLSX)

## Abbreviations

AA: arachidonic acid
AAFCO: Association of American Feed Control Officials
AGE: advanced glycation end product
BUN: blood urea nitrogen
CKD: chronic kidney disease
CBC: complete blood count
Cr: creatinine
DEXA: dual-energy X-ray absorptiometry
DHA: docosahexaenoic acid
EPA: eicosapentaenoic acid
FA: fatty acid
IRIS: International Renal Interest Society
MUFA: monounsaturated fatty acids
PUFA: polyunsaturated fatty acids
scFOS: short chain fructooligosaccharides
SAM: S-adenosylmethionine
SAH: S-adenosylhomocysteine
SDMA: symmetric dimethylarginine
SFA: saturated fatty acids
UPC: urine protein/creatinine ratio
USG: urine specific gravity

## References

[1] Hall JA, Yerramilli M, Obare E, Yerramilli M, Yu S, Jewell DE. Comparison of serum concentrations of symmetric dimethylarginine and creatinine as kidney function biomarkers in healthy geriatric cats fed reduced protein foods enriched with fish oil, L-carnitine, and medium-chain triglycerides. Vet J. 2014;202(3):588–96. Epub 2014/12/03. doi: 10.1016/j.tvjl.2014.10.021 . 25458884

[2] Polzin DJ. Evidence-based step-wise approach to managing chronic kidney disease in dogs and cats. J Vet Emerg Crit Care (San Antonio). 2013;23(2):205–15. Epub 2013/03/09. doi: 10.1111/vec.12034 . 23470210

[3] Wang XH, Mitch WE. Mechanisms of muscle wasting in chronic kidney disease. Nat Rev Nephrol. 2014;10(9):504–16. Epub 2014/07/02. doi: 10.1038/nrneph.2014.112 . 24981816 PMC4269363

[4] Vlassara H, Torreggiani M, Post JB, Zheng F, Uribarri J, Striker GE. Role of oxidants/inflammation in declining renal function in chronic kidney disease and normal aging. Kidney Int Suppl. 2009;(114):S3–11. Epub 2009/12/01. doi: 10.1038/ki.2009.401 . 19946325

[5] Wernimont SM, Radosevich J, Jackson MI, Ephraim E, Badri DV, MacLeay JM, et al. The effects of nutrition on the gastrointestinal microbiome of cats and dogs: Impact on health and disease. Front Microbiol. 2020;11:1266. Epub 2020/07/17. doi: 10.3389/fmicb.2020.01266 ; PMCID: PMC7329990. 32670224 PMC7329990

[6] Koppe L, Fouque D. Microbiota and prebiotics modulation of uremic toxin generation. Panminerva Med. 2017;59(2):173–87. PMID: WOS:000405408700009. doi: 10.23736/S0031-0808.16.03282-1 28001024

[7] Bliss DZ, Stein TP, Schleifer CR, Settle RG. Supplementation with gum arabic fiber increases fecal nitrogen excretion and lowers serum urea nitrogen concentration in chronic renal failure patients consuming a low-protein diet. Am J Clin Nutr. 1996;63(3):392–8. doi: 10.1093/ajcn/63.3.392 . 8602598

[8] Meijers BK, De Preter V, Verbeke K, Vanrenterghem Y, Evenepoel P. p-Cresyl sulfate serum concentrations in haemodialysis patients are reduced by the prebiotic oligofructose-enriched inulin. Nephrol Dial Transplant. 2010;25(1):219–24. Epub 20090819. doi: 10.1093/ndt/gfp414 . 19692415

[9] Sabatino A, Regolisti G, Brusasco I, Cabassi A, Morabito S, Fiaccadori E. Alterations of intestinal barrier and microbiota in chronic kidney disease. Nephrol Dial Transplant. 2015;30(6):924–33. Epub 2014/09/06. doi: 10.1093/ndt/gfu287 . 25190600

[10] Yasuda K, Roneker KR, Miller DD, Welch RM, Lei XG. Supplemental dietary inulin affects the bioavailability of iron in corn and soybean meal to young pigs. J Nutr. 2006;136(12):3033–8. Epub 2006/11/23. doi: 10.1093/jn/136.12.3033 . 17116716

[11] Liu YL, Pan Y, Wang X, Fan CY, Zhu Q, Li JM, et al. Betaine reduces serum uric acid levels and improves kidney function in hyperuricemic mice. Planta Med. 2014;80(1):39–47. doi: 10.1055/s-0033-1360127 . 24338552

[12] McGregor DO, Dellow WJ, Robson RA, Lever M, George PM, Chambers ST. Betaine supplementation decreases post-methionine hyperhomocysteinemia in chronic renal failure. Kidney Int. 2002;61(3):1040–6. doi: 10.1046/j.1523-1755.2002.00199.x . 11849459

[13] Ueland PM, Holm PI, Hustad S. Betaine: A key modulator of one-carbon metabolism and homocysteine status. Clin Chem Lab Med. 2005;43(10):1069–75. doi: 10.1515/CCLM.2005.187 . 16197300

[14] Schwab U, Torronen A, Toppinen L, Alfthan G, Saarinen M, Aro A, et al. Betaine supplementation decreases plasma homocysteine concentrations but does not affect body weight, body composition, or resting energy expenditure in human subjects. Am J Clin Nutr. 2002;76(5):961–7. PMID: WOS:000178913500008. doi: 10.1093/ajcn/76.5.961 12399266

[15] Parnell JA, Reimer RA. Weight loss during oligofructose supplementation is associated with decreased ghrelin and increased peptide YY in overweight and obese adults. Am J Clin Nutr. 2009;89(6):1751–9. Epub 2009/04/24. doi: 10.3945/ajcn.2009.27465 ; PMCID: PMC3827013. 19386741 PMC3827013

[16] Maceyka M, Spiegel S. Sphingolipid metabolites in inflammatory disease. Nature. 2014;510(7503):58–67. doi: 10.1038/nature13475 ; PMCID: PMC4320971. 24899305 PMC4320971

[17] Huang WC, Nagahashi M, Terracina KP, Takabe K. Emerging role of sphingosine-1-phosphate in inflammation, cancer, and lymphangiogenesis. Biomolecules. 2013;3(3). doi: 10.3390/biom3030408 ; PubMed Central PMCID: PMC3839861. 24286034 PMC3839861

[18] NRC. Guide for the Care and Use of Laboratory Animals. Washington, DC: National Academy Press, 2011.

[19] Badri DV, Jackson MI, Jewell DE. Dietary protein and carbohydrate levels affect the gut microbiota and clinical assessment in healthy adult cats. J Nutr. 2021;151(12):3637–50. doi: 10.1093/jn/nxab308 ; PMCID: PMC8643606. 34587256 PMC8643606

[20] International Renal Interest Society. IRIS 2009 Staging of CKD. http://www.iris-kidney.com. Available from: http://www.iris-kidney.com.

[21] Hall JA, Melendez LD, Jewell DE. Using gross energy improves metabolizable energy predictive equations for pet foods whereas undigested protein and fiber content predict stool quality. PLoS One. 2013;8(1):e54405. Epub 2013/01/24. doi: 10.1371/journal.pone.0054405 ; PMCID: PMC3544805. 23342151 PMC3544805

[22] Hall JA, Jackson MI, Vondran JC, Vanchina MA, Jewell DE. Comparison of circulating metabolite concentrations in dogs and cats when allowed to freely choose macronutrient intake. Biol Open. 2018;7(11). doi: 10.1242/bio.036228 ; PMCID: PMC6262854. 30254078 PMC6262854

[23] Zhong Y, Yan Z, Song B, Zheng C, Duan Y, Kong X, et al. Dietary supplementation with betaine or glycine improves the carcass trait, meat quality and lipid metabolism of finishing mini-pigs. Anim Nutr. 2021;7(2):376–83. Epub 20210227. doi: 10.1016/j.aninu.2020.08.010 ; PMCID: PMC8245815. 34258425 PMC8245815

[24] Cholewa JM, Wyszczelska-Rokiel M, Glowacki R, Jakubowski H, Matthews T, Wood R, et al. Effects of betaine on body composition, performance, and homocysteine thiolactone. J Int Soc Sports Nutr. 2013;10(1):39. Epub 20130822. doi: 10.1186/1550-2783-10-39 ; PMCID: PMC3844502. 23967897 PMC3844502

[25] Li Y, Jiang C, Xu G, Wang N, Zhu Y, Tang C, et al. Homocysteine upregulates resistin production from adipocytes in vivo and in vitro. Diabetes. 2008;57(4):817–27. Epub 20080111. doi: 10.2337/db07-0617 . 18192543

[26] Sun CY, Hsu HH, Wu MS. p-Cresol sulfate and indoxyl sulfate induce similar cellular inflammatory gene expressions in cultured proximal renal tubular cells. Nephrol Dial Transplant. 2013;28(1):70–8. Epub 2012/05/23. doi: 10.1093/ndt/gfs133 . 22610984

[27] Fedotova MV. Compatible osmolytes—bioprotectants: Is there a common link between their hydration and their protective action under abiotic stresses? J Mol Liq. 2019;292. ARTN 111339 doi: 10.1016/j.molliq.2019.111339 PubMed PMID: WOS:000488658900002.

[28] Kempson SA, Vovor-Dassu K, Day C. Betaine transport in kidney and liver: Use of betaine in liver injury. Cell Physiol Biochem. 2013;32(7):32–40. Epub 2014/01/17. doi: 10.1159/000356622 . 24429813

[29] Calder PC. Very long-chain n-3 fatty acids and human health: fact, fiction and the future. P Nutr Soc. 2018;77(1):52–72. PMID: WOS:000425963600007. doi: 10.1017/S0029665117003950 29039280

[30] Bauer JE. Therapeutic use of fish oils in companion animals. J Am Vet Med Assoc. 2011;239(11):1441–51. Epub 2011/11/18. doi: 10.2460/javma.239.11.1441 . 22087720

[31] Papasotiriou M, Genovese F, Klinkhammer BM, Kunter U, Nielsen SH, Karsdal MA, et al. Serum and urine markers of collagen degradation reflect renal fibrosis in experimental kidney diseases. Nephrol Dial Transplant. 2015;30(7):1112–21. Epub 2015/03/19. doi: 10.1093/ndt/gfv063 . 25784725

[32] Bohlender JM, Franke S, Stein G, Wolf G. Advanced glycation end products and the kidney. Am J Physiol Renal Physiol. 2005;289(4):F645–59. Epub 2005/09/15. doi: 10.1152/ajprenal.00398.2004 . 16159899

[33] Chaudhuri J, Bains Y, Guha S, Kahn A, Hall D, Bose N, et al. The role of advanced glycation end products in aging and metabolic diseases: Bridging association and causality. Cell Metab. 2018;28(3):337–52. Epub 2018/09/06. doi: 10.1016/j.cmet.2018.08.014 ; PubMed Central PMCID: PMC6355252. 30184484 PMC6355252

